# Nrf2/Keap1-Pathway Activation and Reduced Susceptibility to Chemotherapy Treatment by Acidification in Esophageal Adenocarcinoma Cells

**DOI:** 10.3390/cancers13112806

**Published:** 2021-06-04

**Authors:** Lucie Storz, Philipp Walther, Olga Chemnitzer, Orestis Lyros, Stefan Niebisch, Matthias Mehdorn, Boris Jansen-Winkeln, Yusef Moulla, Thomas Büch, Ines Gockel, René Thieme

**Affiliations:** 1 Department of Visceral, Transplant, Thoracic and Vascular Surgery, University Hospital of Leipzig, Liebigstrasse 20, 04103 Leipzig, Germany; Lucie.Storz@gmx.de (L.S.); philipp.walther@medizin.uni-leipzig.de (P.W.); olga.chemnitzer@medizin.uni-leipzig.de (O.C.); orestis.lyros@medizin.uni-leipzig.de (O.L.); stefan.niebisch@medizin.uni-leipzig.de (S.N.); matthias.mehdorn@medizin.uni-leipzig.de (M.M.); boris.jansen-winkeln@medizin.uni-leipzig.de (B.J.-W.); yusef.moulla@medizin.uni-leipzig.de (Y.M.); ines.gockel@medizin.uni-leipzig.de (I.G.); 2 Medical Faculty, Rudolf-Boehm-Institute for Pharmacology and Toxicology, Clinical Pharmacology, University of Leipzig, Haertelstrasse 16–18, 04103 Leipzig, Germany; thomas.buech@medizin.uni-leipzig.de

**Keywords:** Barrett’s esophagus, esophageal adenocarcinoma, chemotherapy, gastroesophageal reflux disease (GERD), inflammation

## Abstract

**Simple Summary:**

Inflammation caused by acidic reflux contributes to disease progression in Barrett’s esophagus. Little is known, whether esophageal cancer cells are influenced by acidic reflux and whether reflux influences cancer cell physiology, targeting the Nrf2/Kepa1- and the NFκB-pathway. The understanding mechanisms of the acidic susceptibility in cells from advanced stages of Barrett’s esophagus will provide further evidence, whether it should be prevented during chemotherapy for EAC treatment.

**Abstract:**

Chronic acid reflux causes cellular damage and inflammation in the lower esophagus. Due to these irritating insults, the squamous epithelium is replaced by metaplastic epithelium, which is a risk factor for the development of esophageal adenocarcinoma (EAC). In this study, we investigated the acid susceptibility in a Barrett’s cell culture in vitro model, using six cell lines, derived from squamous epithelium (EPC1 and EPC2), metaplasia (CP-A), dysplasia (CP-B), and EAC (OE33 and OE19) cells. Cells exposed to acidic pH showed a decreased viability dependent on time, pH, and progression status in the Barrett’s sequence, with the highest acid susceptibility in the squamous epithelium (EPC1 and EPC2), and the lowest in EAC cells. Acid pulsing was accompanied with an activation of the Nrf2/Keap1- and the NFκB-pathway, resulting in an increased expression of HO1—independent of the cellular context. OE33 showed a decreased responsiveness towards 5-FU, when the cells were grown in acidic conditions (pH 6 and pH 5.5). Our findings suggest a strong damage of squamous epithelium by gastroesophageal reflux, while Barrett’s dysplasia and EAC cells apparently exert acid-protective features, which lead to a cellular resistance against acid reflux.

## 1. Introduction

The incidence of esophageal adenocarcinoma (EAC) in the Western world has increased during the last decades. Currently, esophagectomy is the only curative treatment for the majority of cases beyond early tumor stages (T1a, N0) [1]. Patients with locally advanced tumor stages have to undergo additional perioperative chemotherapy with FLOT [2] and achieve an improved overall survival of 50 months with a 3-year survival rate of 57% [3]. Currently, it is investigated, whether patients with esophageal adenocarcinoma will profit from radiochemotherapy according to the CROSS protocol compared to chemotherapy with the FLOT protocol (ESOPEC trial) [4]. Response to chemotherapy varies between individuals quite largely, and, so far, reliable data about predictive factors are limited [5,6]. The presence of gastroesophageal reflux disease (GERD) during neoadjuvant chemotherapy might be relevant for the success of treatment regimes.

In most cases, EAC derives from metaplasia with intestinal metaplasia, the Barrett’s esophagus (BE), which can progress to high-grade intraepithelial neoplasia (HGIN) and invasive adenocarcinoma [1,7]. Chronic gastroesophageal reflux disease (GERD) is likely to be the leading risk factor for BE, causing chronic inflammation through acid and bile exposure of the esophageal mucosa [1,8,9]. About 10% of patients suffering from chronic GERD develop BE. The risk of EAC development in BE patients is 0.10 to 0.15% per year [10]. However, it is still unclear, which exact molecular mechanisms are triggered by GERD. The detailed mechanisms, which affect the esophageal mucosa, leading to progression of inflammation and metaplasia, have not completely been clarified yet. The impact of tissue damage through chronic inflammation at different stages of BE and EAC has to be explained, and the involved pathways are still largely unknown. Suitable in vitro models will relevantly help to address these goals.

Both transcription factors, Nrf2 and NFκB, could play an important role in the development and progression of BE [11,12]. Nrf2 is released from its inhibitor Keap1 due to cellular or oxidative stress and binds to the antioxidant responsive element (ARE) [13]. This leads to an increased transcription of cytoprotective genes including glutathione reductase, glutathione peroxidase 2, thioredoxin reductase 1, glucose-6-phosphate dehydrogenase, and Hemoxygenase-1 (HO1), which can be used as an indicator of elevated Nrf2 activity [13]. NFκB is a transcription factor involved in regulation of cell cycle control, apoptosis, cellular stress, inflammation, and immune response [12]. Overexpression of NFκB has been found in different types of tumors, including EAC [6]. According to recent studies, NFκB may serve as a predictor for neoadjuvant chemotherapy response and survival of patients with EAC: low expression is statistically associated with a better response and improved survival [6]. Furthermore, the NFκB activity detected in biopsy specimens in untreated patients was higher than in specimens received post-chemotherapy in the same patients [14]. To analyze the acid susceptibility and the NFκB and Nrf2/Keap1-pathway activation, we will attract attention to cellular viability due to inflammation and potential activation of the Nrf2/Keap1-pathway, resulting in resistance to chemotherapy in a Barrett’s esophagus in vitro model and an organotypic 3D-cultures system.

## 2. Materials and Methods

### 2.1. Cell Culture

**Figure 1:**
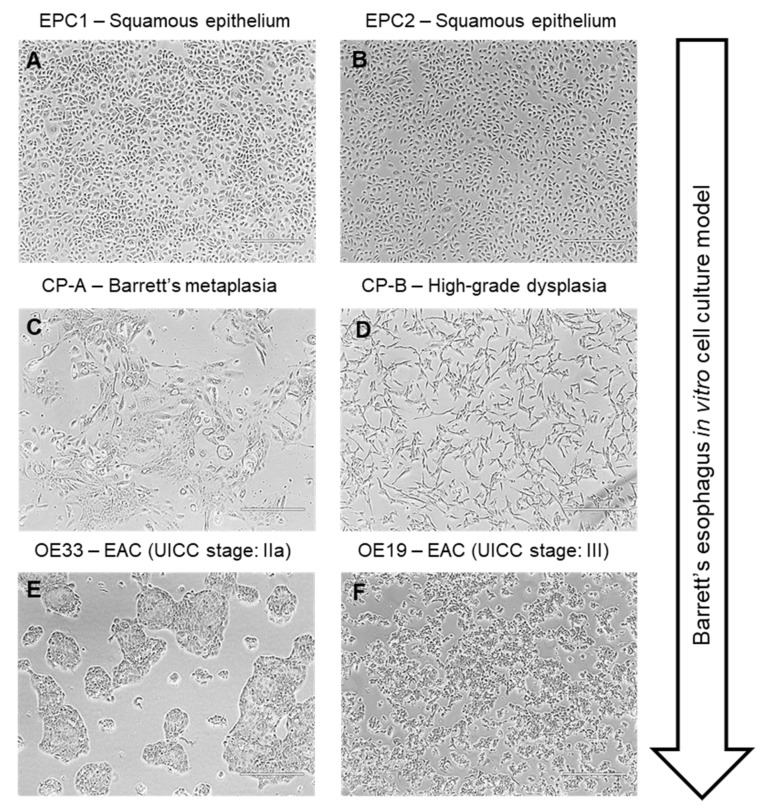
The Barrett’s in vitro cell culture model consistent of the two squamous epithelium cell lines EPC1 (**A**) and EPC2 (**B**), the metaplastic cell line CP-A (**C**), the high-grade dysplastic cell line CP-B (**D**), and the two EAC cell lines OE33 (**E**) and OE19 (**F**).

Six different immortalized human esophageal cell lines and one fibroblast cell line were grown in monolayer cell culture at 37 °C with 5% CO2. The six cell lines represented different stages of Barrett’s esophagus to EAC in a Barrett’s esophagus in vitro cell culture model (Figure 1A–E).

EPC1-hTERT and EPC2-hTERT squamous esophageal cells were a generous gift from Dr. Hiroshi Nakagawa [15] and were cultivated in KSFM growth medium (Thermo Fisher, Darmstadt, Germany), as previously described [16]. EPC1-hTERT and EPC2-hTERT were immortalized normal human esophageal epithelial cells to overcome senescence. The metaplastic cell line CP-A (CRL-4027) and the high-grade dysplastic cell line CP-B (CRL-4028) were purchased from LGC Standards (Wesel, Germany) and cultured according to the manufacturer’s protocol, using MCDB 153 growth medium (Biochrom, Berlin, Germany). Esophageal adenocarcinoma cells OE33 (ECACC-96070808) and OE19 (ECACC-96071721) (Sigma-Aldrich, Taufkirchen, Germany) were cultured in RPMI-1640 medium (ThermoFisher, Darmstadt, Germany) supplemented with 10% fetal bovine serum (Sigma-Aldrich, Taufkirchen, Germany) [17]. The fetal human esophageal fibroblast cell line FEF3 [15] was cultured in DMEM (ThermoFisher, Darmstadt, Germany) supplemented with 10% fetal bovine serum.

### 2.2. Treatment with Acidified Medium

A total of 250,000 cells were seeded in a 6-well plate in 2 mL medium and grown for 24 h. Medium was replaced by 2 mL acidified medium. To adjust the pH-value of the respective medium, 6.0 M HCl (CarlRoth, Karlsruhe, Germany) was used to titrate the required pH of pH 6, pH 5, pH 4, pH 3.5, or pH 3. The acidified medium was strained through a sterile filter. After the required treatment, medium was removed, and cells were washed with 2 mL of ice cold PBS. To simulate repetitive acidic reflux, EPC1, EPC2, CP-A, and CP-B cells were treated three times (10 min) for two days. All cells were harvested for RNA or protein extraction directly after the last treatment.

### 2.3. Protein Isolation and Western Blotting

The cells were lysed in RIPA buffer and protein concentration was measured using Bradford’s method. Nuclear proteins were isolated as previously described [18]. Briefly, the residual pallet was solubilized in 100 µL NLB buffer (50 mM Tris (pH 8.1), 10 mM ETDA, 1% SDS). Afterwards, samples were sonicated (Bioruptor, Diagenode, Seraing, Belgium). Further, 20 µg of total and nucleus protein were separated on 8% or 12% sodium dodecyl sulfate (SDS)-polyacrylamide gels and blotted on nitrocellulose membranes. Protein transfer was validated using Ponceau-S staining. The membranes were blocked with 5% low fat milk for 1 h and incubated with the specific primary antibody at 4 °C overnight (Table 1). A peroxidase conjugated secondary antibody was used to detect the primary antibody and incubated at room temperature for 1 h. Protein bands were visualized with ECL chemiluminescence detection reagent (Millipore, Billerica, MA, USA), which were analyzed densitometrically using ImageJ [19].

### 2.4. RNA Isolation and cDNA Synthesis

Total RNA was isolated using the RNeasy Plus Mini Kit (Qiagen, Hilden, Germany) according to the manufacturer’s protocol. cDNAs were synthesized from 500 ng of total RNA using the RevertAid RT synthesis kit (Thermo Scientific, Darmstadt, Germany).

### 2.5. Quantitative RT-PCR

Quantitative RT-PCR was performed with the SYBR Green JumpStart Taq ReadyMix Kit (Sigma-Aldrich, Taufkirchen, Germany; RotorGene RG-3000, Qiagen, Hilden, Germany). All samples were run in duplicates. Β-Actin was used for normalization (Table 2).

### 2.6. Viability Assay

Viability assays were performed in 96-well plates. Then, 3500 cells per well were seeded and grown for 72 h. Medium was replaced with acidified medium at pH 4, pH 3.5, or pH 3. Full culture medium served as control. The acidified medium was removed after 15 or 30 min. The viability was assessed with PrestoBlue Cell Viability Reagent stain (ThermoFisher, Darmstadt, Germany) and subsequent absorbance measurement with SpectraMax M5 (Molecular Devices, Sunnyvale, CA, USA).

In addition, 5-FU (Pharmacological preparation, Pharmacy, University Hospital of Leipzig) susceptibility was investigated in OE33 and OE19 cells. Further, 5000 cells were seeded in 96-well plates and grown for 24 h. Medium was replaced with acidified medium (pH 6 and pH 5.5), containing 0.6 µM 5-FU for OE33 and 5 µM for OE19 cells for 24 h.

### 2.7. ARE Luciferase Assay

ARE activity was conducted with 20,000 cells, seeded in white 96-well plates 24 h before transfection (Greiner, Frickenhausen, Germany). Transfection was performed with 100 ng plasmid (ARE Reporter Kit, Amsbio, Abingdon, UK) and 0.3 µL FuGENE (Promega, Walldorf, Germany) in a ratio 3:1 for 24 h. Cells were treated with acidified medium (pH 4) for 15 and 30 min, respectively. Afterwards, cells were grown for 24 h with neutral medium. The activity of the reporter assay was determined by Luciferase Dual Glo (Promega, Walldorf, Germany) according to the manufacturer’s protocol.

### 2.8. 3D-Culture

Organotypic 3D-cultures (OTC) were grown in 12-well plates as described previously [20]. Briefly, FEF3 were embedded in matrigel and used for the stromal compartment of the OTCs (Corning, Amsterdam, NL, USA). EPC2 were seeded to form the epithelial layer on day 6, and an air–liquid interface was performed to receive a stratified epithelium. Exposure with acidic growth medium (pH 4) was done in the top well at day 20 and was performed for one minute three times a day on five consecutive days. Four hours after the last exposure, the OTCs were washed with PBS three times, submerged in 4% paraformaldehyde for 3 h, and left in 70% ethanol overnight before embedding them in paraffin.

### 2.9. Histology and Immunohistochemistry

The paraffin embedded 3D-cultures were cut in 3 µm sections. For hematoxylin-eosin (HE) staining, sections were stained with hematoxylin for 6 min and rinsed with tap water for 6 min. After short rinsing with distilled water, eosin staining was performed for 2 min.

### 2.10. Immunohistochemistry for HO1

After deparaffinization, the sections were washed with PBST. A 3% H2O2/MeOH solution was used to block endogenous peroxidase enzymes at 4 °C for 20 min. For blocking unspecific antibody bindings, sections were covered with 5% NormalGoatSerum (Jackson Immuno Research, Suffolk, UK) at room temperature for 20 min and washed with PBST. Incubation with the primary antibody (Table 2) was done at 4 °C overnight, washed 5 times with PBST, incubated with a secondary biotin conjugated antibody and a peroxidase conjugated streptavidin (Jackson Immuno Research, Suffolk, UK), each at room temperature for 60 min (diluted 1:1000 in 1% BSA in PBS). Visualization of the substrate was achieved with diaminobenzidine for 4 min, followed by a counterstaining with hematoxylin. Areas of high expression appear in a dark brown.

### 2.11. Statistics

Analyses were performed using GraphPad Prism 6 (GraphPad Software Inc., San Diego, CA, USA). Results are presented as mean ± standard error (S.E.M.). A *p*-value of less than 0.05 was considered statistically significant. The statistical tests used are provided in the corresponding figure legends.

## 3. Results

### 3.1. Cellular Viability in Cells of the Barrett’s Sequence by a Reflux-Like Acidic Exposure

**Figure 2:**
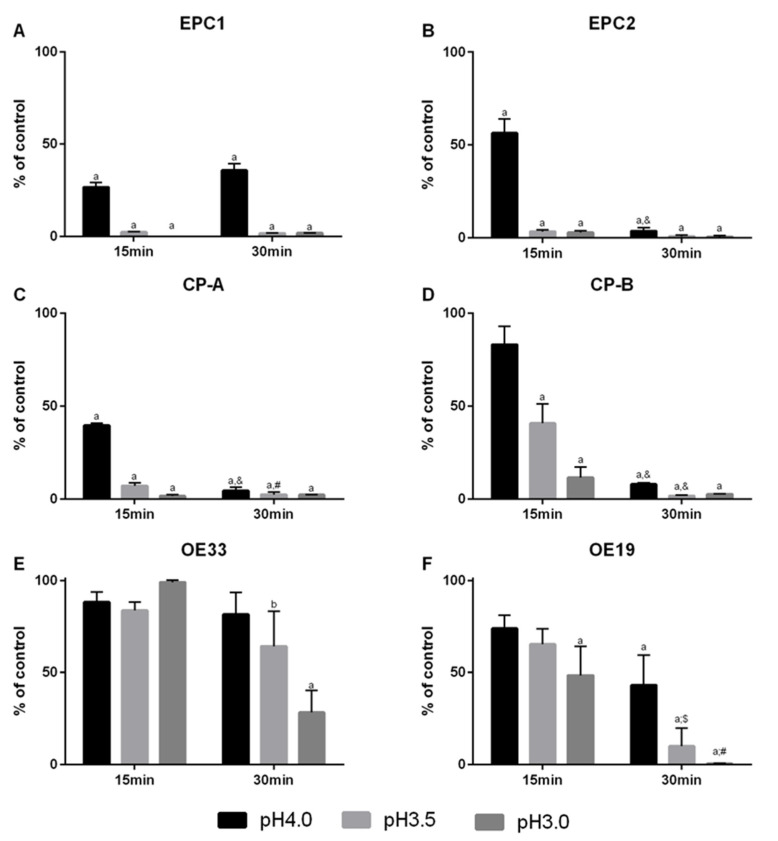
Acid susceptibility in a Barrett’s esophagus cell culture model. Esophageal squamous epithelium cell lines EPC1 (**A**), EPC2 (**B**); Barrett’s metaplasia cell line CP-A (**C**), Barrett’s high-grade dysplasia cell line CP-B (**D**), and esophageal adenocarcinoma cell lines OE33 (**E**) and OE19 (**F**) were exposed to acidified media with pH 4, pH 3.5, or pH 3 for 15 and 30 min. Full growth medium (pH 7.4) served as controls. Viability was measured photometrically by PrestoBlue Cell Viability Reagent stain. Values are shown as mean ± S.E.M. (Two-way ANOVA with Sidak’s multiple comparisons test; a—*p* < 0.001, b—*p* < 0.01 compared to control; &—*p* < 0.001, $—*p* < 0.01, #—*p* < 0.05, compared to 15 min).

We determined the effect of an acidified medium to two epithelial cell lines (EPC1 and EPC2), a metaplastic cell line (CP-A), a dysplastic cell line (CP-B), and two EAC cell lines (OE33 and OE19). Cellular viability was revealed using acidified medium at pH 4, pH 3.5, and pH 3 and two different exposure times (15 or 30 min) to mimic acid reflux and was compared to the individual growth pattern of each cell lines. Thereby, the cellular viability was time- and pH-value-dependent. Of note, cells from more advanced stages of the Barrett’s sequence, showed lower acid susceptibility than cells from earlier stages. The epithelial cell lines (EPC1 and EPC2) and the metaplastic cell lines (CP-A) showed a loss of approx. 50–75% in cellular viability already in acidified medium at pH 4 after 15 min (Figure 2A–C). No additional loss in cellular viability was observed in EPC1 after 30 min, while EPC2 and CP-A responded with an additional significant loss in cellular viability down to 0–2.5% as compared to control cells. The dysplastic cell line CP-B only showed a significant loss in cellular viability by medium acidified to pH 3.5 and pH 3.0 after 15 min or by a longer treatment of 30 min (Figure 2D). The highest acid tolerability occurred in the EAC cell lines OE33 and OE19. OE33 had a significant loss in cellular viability in cells treated for 30 min with pH 3.5 and pH 3.0, but without reaching a bottom height of EPC1, EPC2, CP-A, and CP-B cells (Figure 2E). However, OE19 cells showed a significant loss in cellular viability in pH 3.0 treated cells for 15 min and in pH 4, pH 3.5, and pH 3.0 treated cells after 30 min (Figure 2F). OE33 cells displayed the highest robustness against low pH-values.

### 3.2. Nrf2 and HO1 Expression in EAC Cells

**Figure 3:**
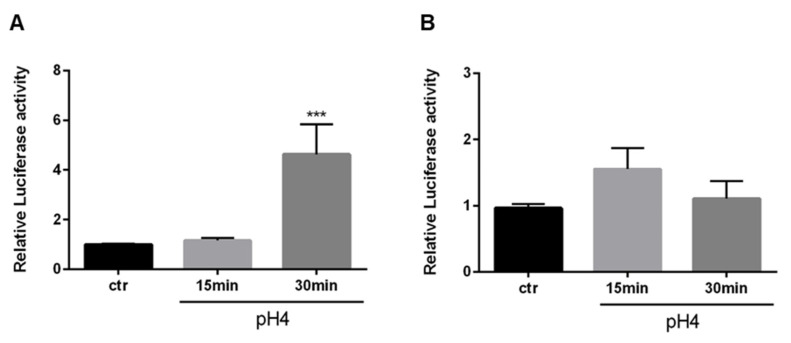
Acidified medium induces Nrf2 activity in a cell line specific manner. OE33 (**A**) and OE19 (**B**) were treated with acidified medium (pH 4) for 15 and 30 min and were analyzed by luciferase assay. Values are shown as mean ± S.E.M. (Kruskal–Wallis test with Dunn’s multiple comparisons test; ***—*p* < 0.001, compared to untreated controls).

It was investigated, whether acid exposure induces Nrf2-mediated transcription activity at pH 4, which was chosen to exclude cellular damage, which was low in EAC cells at pH 4. By luciferase assay, a peak of significant NRF2-activation in OE33 was shown after acid exposure for 30 min (Figure 3A). However, there was no additional NRF2-activation observed by luciferase assay in OE19 cells (Figure 3B).

**Figure 4:**
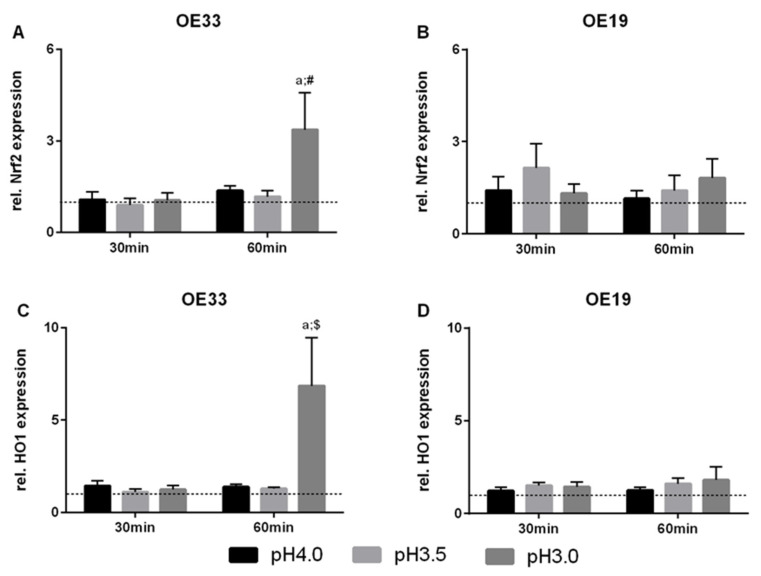
Gene expression in EAC cells after acute acid exposure. Expression of Nrf2 (**A**,**B**) and HO1 (**C**,**D**) were assessed via quantitative RT-PCR. OE33 and OE19 cells were treated once (acute) with acidic medium for 30 and 60 min. Values are shown as mean ± S.E.M. (Two-way ANOVA with Sidak’s multiple comparisons test; a—*p* < 0.001, compared to control; $—*p* < 0.01, #—*p* < 0.05, compared to 15 min).

Furthermore, distinct expression of Nrf2 and HO1 were investigated. Nrf2 expression levels were significantly elevated (3.4-fold) in OE33 cells following exposure to pH 3.0 medium for 30 min (Figure 4A). OE19 cells did not show any change in Nrf2 expression levels (Figure 4B). In line with these results, OE33 cells displayed an increase in HO1 expression (6.9-fold), OE19 did not, compared to control cells (Figure 4C,D).

### 3.3. pH 4-Induced Nrf2 and NFκB Activation in EAC Cells

**Figure 5:**
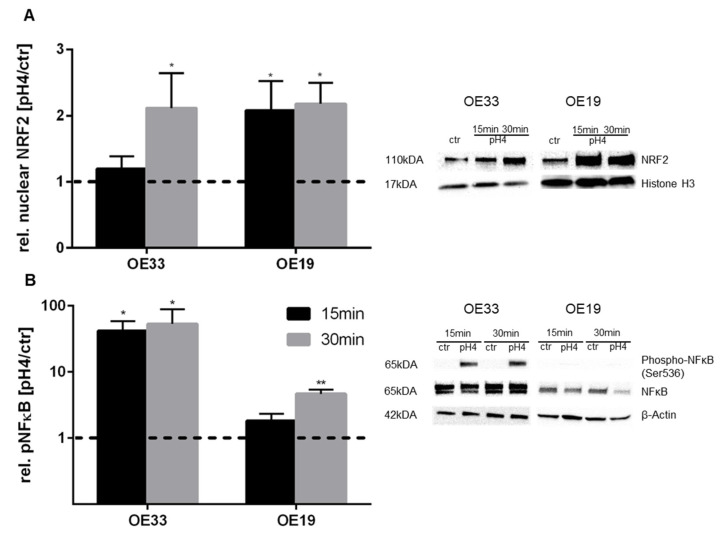
NRF2 and NFκB phosphorylation in EAC cells after acute acid exposure. Nuclear accumulation of NRF2 (**A**) and the phosphorylation of NFκB (**B**) were investigated. Both, nuclear NRF2 and NFκB phosphorylation were measured by Western blot. OE33 and OE19 cells were treated once with acidic medium (pH 4) for 15 and 30 min. A representative Western blot is shown for NRF2 and NFκB. Values are shown as mean ± S.E.M. (Kruskal–Wallis test with Dunn’s multiple comparisons test; **—*p* < 0.01, *—*p* < 0.05 compared to untreated controls).

To show NRF2 activation, its nuclear localization was analyzed in EAC cells by Western blot. After exposure of OE33 and OE19 cells for 15 and 30 min, the nuclear protein fraction was analyzed. Thereby, OE33 showed an increase of nuclear NRF2 by 2.1-fold only after 30 min exposure to acidified medium, while OE19 revealed an earlier response, yet after 15 min and 30 min by 2.1-fold and 2.2-fold compared to controls (dashed line), respectively (Figure 5A).

Since NFκB activity has already been shown to be higher in BE and EAC compared to healthy tissues of the same patient, we investigated the impact of acid-induced NFκB phosphorylation in EAC cells by Western blot. The EAC cell lines OE33 and OE19 were treated with medium at pH 4 for 15 or 30 min, which induced significant higher levels of NFκB phosphorylation in both cell lines. However, OE33 cells showed a significant higher phosphorylation of NFκB, than OE19 cells, independent of the exposure time. OE19 cells displayed a 4.7-fold increase in NFκB phosphorylation only after 30 min (Figure 5B).

### 3.4. Dependency of 5-FU Effectiveness with Regard to a pH-Shift

**Figure 6:**
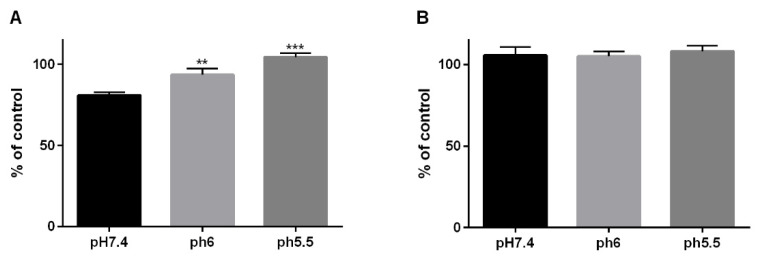
5-FU susceptibility against acidic pH. OE33 (**A**) and OE19 (**B**) cells were treated with 10 µM, respectively, 5-FU at pH 7.4 (neutral) or in acidified medium at pH 6 and pH 5.5 for 24 h. Values are shown as mean ± S.E.M (One-way ANOVA with Holm–Sidak’s multiple comparisons test; ***—*p* < 0.001, **—*p* < 0.01 compared to controls).

OE33 and OE19 cells were treated with 10 µM 5-FU, respectively. Additionally, the cells were grown in medium at pH 6 or pH 5.5, and full growth medium served as control (pH 7.4). The cells were grown in acidified medium of pH 6 and pH 5.5, as 5-FU treatment takes longer time intervals and the cells do not tolerate lower pH-values for longer than 30 min. 5-FU treatment resulted in a decreased cellular viability of OE33 cells, when cultured in neutral (pH 7.4) culture medium, while the usage of acidified growth medium at pH 6 and pH 5.5 did not result in a reduced viability compared to the corresponding control (Figure 6A). In OE19 cells, no change in cellular viability was observed, using neutral or acidified culture medium by 5-FU at 10 µM (Figure 6B).

### 3.5. Organotypic 3D-Cultures and Nrf2 and HO1 Expression in Non-Malignant Cells with Chronical Acid Exposure

**Figure 7:**
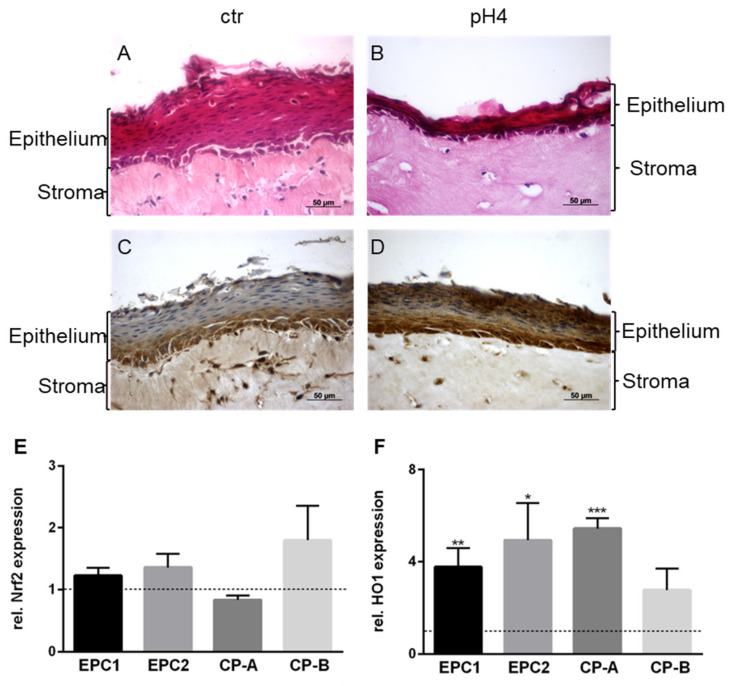
Histological and immunohistochemical staining of organotypic 3D-cultures and gene expression in non-malignant cells with chronical acid treatment. The squamous epithelial cell line (EPC2) was grown on top of a matrix-embedded fibroblasts (FEF3) layer in organotypic 3D-cultures and treated with acidic medium (pH 4) chronically for five consecutive days and three times a day for 1 min. Sections were stained with hematoxylin-eosin (**A**,**B**) and immunohistochemistry (DAB) was done for HO1 (**C**,**D**). The organotypic 3D-cultures are shown in 40× magnification. Expression of Nrf2 (**E**) and HO1 (**F**) were assessed via quantitative RT-PCR. EPC1 and EPC2 (esophageal squamous epithelium), CP-A (Barrett’s metaplasia) and CP-B (Barrett’s high-grade dysplasia) were treated two days. Values are shown as mean ± S.E.M. (Student’s t-test was performed compared to the respective untreated control; ***—*p* < 0.001, **—*p* < 0.01, *—*p* < 0.05).

To investigate, whether Nrf2 and HO1 are affected in non malignant cells after chronically acid exposure, esophageal organotypic 3D-cultures (OTC) were carried out using EPC2 cells. Additionally, the mRNA expression of Nrf2 and HO1 were investigated in EPC1, EPC2, CP-A, and CP-B cells. EPC2 cells constitute a stratified squamous epithelium in a crosstalk with matrigel-embedded esophageal stromal fibroblasts (FEF3). To mimic acid reflux, the OTCs were treated with acidified medium (pH 4) from day 20–25. Acidic exposure led to shrinkage of the epithelial layer (Figure 7A,B). Expression levels of HO1 were analyzed by immunohistochemistry. HO1 immunohistochemistry demonstrated an increased presence of the enzyme in the pH 4-exposed samples (Figure 7C,D). Since transcription factor Nrf2 and its downstream target HO1 play an important role in inflammation and self-protection of cells against cellular stress, we determined their gene expression after repeated exposure to acidified medium. To mimic recurring reflux, EPC1, EPC2, CP-A, and CP-B were treated three times per day with pH 4 media for two consecutive days. While Nrf2 expression levels were not significantly altered in the four cell lines (Figure 7E), HO1 expression was 3.8-fold, 4.9-fold, 5.4-fold, and 2.8-fold increased in EPC1, EPC2, CP-A, and CP-B, respectively, compared to control cells (Figure 7F).

## 4. Discussion

In this study, we clearly showed that cells from more advanced stages of the Barrett’s sequence have a higher resistance to an acidic pH-shift. Moreover, mimicking reflux in vitro led to an activation of the Nrf2/Keap1- and NFκB-pathway. The EAC cells OE33 and OE19 were characterized by a reduced acid susceptibility compared to the epithelial cell lines EPC1 and EPC2, the metaplastic cell line CP-A and the dysplastic cell line CP-B. The activation of the Nrf2/Keap1-pathway was present in all cell lines investigated, except for OE19, which was shown by nuclear translocation of Nrf2 in both EAC cell lines and an increased luciferase activity in OE33 cells. In an organotypic 3D-culture system, we could confirm the damage of the stratified squamous epithelium by acidic pH and the induction of the Nrf2/Keap1-pathway downstream molecule HO1. Finally, we demonstrated an induced inflammation by acidic pH-values in OE33 and OE19 cells due to a phosphorylation of NFκB and an associated decrease susceptibility to 5-FU in OE33 cells.

Compared to healthy esophageal tissue, the gene expression of Dickkopf 1 (Dkk1) has been shown to be elevated in Barrett’s esophagus. It inhibits the Wnt-pathway through binding LRP6 and is associated with tissue damage, cellular stress, and inflammation. Dkk1 has been elevated in EPC2 cells after the treatment with acidic medium [21].

The EAC cells acquired a higher acid tolerability compared to the squamous epithelium, which might be due to a more robust endowment of cell protective proteins and compounds [22,23,24]. The used pH from 3.0–6.5 do not reflect the physiological conditions of pH 2. However, the chosen condition approximates these conditions, as the cells from the Barrett’s esophagus cell culture model do not tolerate a pH <3.0 for more than 30 min.

Induction of the Nrf2/Keap1- and the NFκB-pathway has been linked to cellular stressors to overcome these insults and to promote survival [13]. The expression of the Nrf2 downstream target HO1 was elevated after two days of intermitted exposure to acidic medium, mimicking repeated reflux events. Treatment with ursodeoxycholic acid thereby prevents deoxycholic acid induced oxidative DNA damage by activating Nrf2 in vitro and in vivo [25]. These findings suggest that antioxidant agents could be used to prevent progression in the Barrett’s sequence. Recently, we have shown a decreased Akt/PI3K-pathway activation and apoptosis induction in OE33 and OE19 cells by curcumin [17]. The Nrf2-mediated antioxidant targets were induced by binding of Nrf2 to promotor regions containing an antioxidant response element (ARE) [26]. In steady-state, Nrf2 is sequestered and bound to Keap1, that is released after Nrf2 phosphorylation, which enables Nrf2 to translocate to the nucleus [27]. In EAC (OE33) cells, we found that Nrf2 and HO1 were increased upon exposure to acidified medium; others had shown an Nrf2-activation by acidic bile salts to overcome GERD induced oxidative stress and DNA double strand breaks [28]. Additionally, we were able to show a nuclear accumulation of Nrf2 after treatment with acidified medium, as well as an increased luciferase activity under ARE control. While Nrf2 mutations are not frequent in EAC, Nrf2 represents a potential durable intracellular molecule [29]. While there is a NRF2-activation in Barrett’s esophagus and EAC, so far, no clinical relevant NRF2-inhibitor is available [30].

Inflammation is involved in the progression of Barrett’s esophagus and especially in EAC by an increased TNFα and TNFR1 signature [31]. NFκB was shown to be constitutively activated in esophageal adenocarcinoma cells [14]. However, the two investigated EAC cell lines showed a different response to acidic pH, resulting in a higher increase in NFκB phosphorylation and luciferase activity in OE33 than in OE19 cells. This might reflect the inter-patient heterogeneity and therefore the limitation of analysis of cell culture systems. Nevertheless, the Barrett’s cell culture model used here represents all stages of the Barrett’s sequence and therefore is a very valuable model to investigate Barrett’s esophagus and Barrett’s carcinoma in vitro [17]. Treating EAC cells with 5-FU, which is a standard chemotherapeutic for EAC patients treated by FLOT [2], has shown a decrease in cellular viability in OE33 cells, while treatment with acidified conditions (pH 6 and pH 5.5) resulted in a decay of the 5-FU-mediated loss of cellular viability. However, OE19 appeared to be 5-FU-resistant. Important mechanisms for chemotherapy resistance to 5-FU and cisplatin are autophagy and inactivation of apoptosis [32]. While OE33 cells undergo apoptosis, OE19 cells do not, when treated by 30 µM 5-FU. Cisplatin also showed a higher loss of viability in OE33 than in OE19 cells [33]. Additionally, OE19 cells are more likely to recover after 5-FU and cisplatin treatment, shown in their capacity to have higher colony numbers in a colony-forming assay after drug treatment [32]. However, docetaxel was investigated only in the EAC cell lines SKGT-4, BE3, and FLO-1, which were more sensitive to docetaxel than OAPC and JHESO cells [34]. To avoid neoplastic progression of Barrett’s esophagus, the use of proton pump inhibitors (PPI) is recommended [35]. Barrett’s patients showed decreased inflammation and proliferation in metaplastic lesions, when treated with PPIs [36]. Thus, the effect of 5-FU treatment to EAC cells in an inflamed environment must be investigated in further studies. Preventing acid reflux in Barrett’s esophagus patients by PPI is also beneficial in EAC patients. Nevertheless, our findings point out the importance of an adequate PPI treatment. EAC patients often (40%) do not have any reflux anamnesis and do not show reflux-associated symptoms [37,38]. These patients are not well covered by the current GERD-symptoms-driven BE and EAC screening [39]. However, an effective control of both, acid and non-acid exposures, is needed, as the impact of bile acids in Barrett’s mucosa has not adequately delineated [40]. Bile acids were found to increase the CDX2 expression, which together with NFκB, is responsible for inflammation in esophageal tissue [41]. CDX2 induction is one of the key events in the development of the Barrett’s esophagus to direct intestinal epithelium [42,43], while acidic and bile acids are able to increase the Cdx2 promotor activity [44,45,46]. Acetylsalicylic acid was shown to inhibit the acid and bile salt induced CDX2 activation and might be therefore a potential drug to prevent the GERD-driven development of Barrett’s esophagus [47].

Organotypic 3D-cultures are characterized by a proliferative basal layer, which was found HO1-positive in controls, while the differentiated luminal cells were HO1-negative. In pH 4 treated cells, both compartments, basal and luminal cells, were found HO1-positive. Organotypic 3D-cultures might be a superior model for further investigation in this field, because they are similar to the original tissue and can be grown under regular cell culture conditions [20]. They can help to understand the interaction of fibroblasts and epithelial cells. Additionally, it may be possible to use primary cells for the epithelial layer, derived from patients suffering from BE or EAC.

## 5. Conclusions

In conclusion, we showed a reduced acid susceptibility in cells from advanced stages of Barrett’s esophagus, with the highest acid protective capacity in esophageal adenocarcinoma cells in a Barrett’s esophagus in vitro model. Acidified conditions induced the Nrf2/Keap1- and the NFκB-pathway. For the first time it was shown, that decreased extracellular pH-values could foil chemotherapeutic effects of 5-FU in vitro. These data should guide further work on suitable in vitro models to address cellular stress response pathways in order to understand the progression of Barrett’s esophagus to a malignant disease and to understand the impact of acidic reflux in tumor cell survival and progression. PPIs itself could reduce tumor cell growth or could indirectly contribute to overcome chemotherapy resistance in EAC patients by the restoration of a neutral esophageal pH. The overall aim must be an effective and successful treatment of EAC and an early detection of patients at high risk for EAC development. These two achievements are long-term strategies to enhance patients’ overall survival.

## Figures and Tables

**Table 1:** Antibodies.

Primary Antibodies for Westernblot Analysis	Dilution	Company	Catalogue Number
β-Actin	1:2500 (0.5% LFM/PBS)	Sigma-Aldrich, Taufkirchen, Germany	A1978
NF-κB p65	1:1000 (1% BSA/TBST)	Cell Signaling Technology, Danvers, USA	8242
Phospho-NFκB p65 (Ser536)	1:1000 (1% BSA/TBST)	Cell Signaling Technology, Danvers, USA	3033
Nrf2	1:1000 (1% BSA/TBST)	Abnova, Taipeh, Taiwan	MAB20252
Histone H3	1:1000 (1% BSA/TBST)	Cell Signaling Technology, Danvers, USA	4499S
**Secondary antibodies for Westernblot analysis**			
Goat anti-rabbit	1:7500 (0.5% LFM)	Jackson Immuno Research, Suffolk, UK	111-035-045
Goat anti-mouse	1:7500 (0.5% LFM).	Jackson Immuno Research, Suffolk, UK	115-035-068
**Primary antibodies for** **Immunohistochemistry**			
HO1	1:100 (1% BSA/PBS)	GeneTex, Inc., Irvine, USA	GTX101147
**Secondary antibodies for Immunohistochemistry**			
Goat anti-rabbit	1:500 (1% BSA/PBS)	Jackson Immuno Research, Suffolk, UK	111-035-045

BSA—Bovine serum albumin; HO1—Heme oxygenase 1; LFM—Low fat milk; NF-κB—nuclear factor kappa-light-chain-enhancer of activated B cells; PBS—Phosphate-buffered saline; TBST—Tris-buffered saline.

**Table 2:** Oligo sequences.

Name	Forward	Reverse	Reference	Length (bp)
β-Actin	GTCTTCCCCTCCATCGTG	AGGGTGAGGATGCCTCTCTT	NM_001101.3	113
NRF2	TCTTGCCTCCAAAGTATGTCAA	ACACGGTCCACAGCTCATC	NM_006164	99
HO1	GAGTGTAAGGACCCATCGGA	GCCAGCAACAAAGTGCAAG	NM_002133	105

bp—base pairs; HO1—Heme oxygenase 1; NRF2—Nuclear factor erythroid 2-related factor 2.

## Data Availability

The datasets generated during the current study are available from the corresponding author on reasonable request.
